# Adverse Drug Reaction Trajectories in Older Adults: From Pharmacological Vulnerability to Clinical Complexity

**DOI:** 10.3390/ijerph23070849

**Published:** 2026-06-29

**Authors:** Fulvio Lauretani, Crescenzo Testa, Marco Salvi, Irene Zucchini, Aurora Merolla, Patrizia Rovere-Querini, Marcello Maggio

**Affiliations:** 1 Geriatric Clinic Unit, University-Hospital of Parma, 43124 Parma, Italy; marco.salvi@unipr.it (M.S.); irene.zucchini@unipr.it (I.Z.); marcellogiuseppe.maggio@unipr.it (M.M.); 2 Department of Medicine and Surgery, University of Parma, 43124 Parma, Italy; 3 Interdepartmental Centre for Sleep Medicine, University of Parma, 43124 Parma, Italy; 4 Vita-Salute San Raffaele University, 20100 Milan, Italy; merolla.aurora@hsr.it (A.M.); rovere.patrizia@hsr.it (P.R.-Q.); 5 Department of Medicine, IRCCS San Raffaele Hospital, 20100 Milan, Italy

**Keywords:** adverse drug reactions, polypharmacy, frailty, older adults, deprescribing, pharmacokinetics, prescribing cascade, anticholinergic burden, medication review, geriatric pharmacology

## Abstract

**Highlights:**

**Public health relevance—How does this work relate to a public health issue?**

- Adverse drug reactions (ADRs) are a major and often preventable cause of hospitalisation, emergency department visits, falls, delirium, and functional decline in the rapidly growing population of older adults living with multimorbidity and polypharmacy.
- Approximately 5% of all hospital admissions are attributable to ADRs, and adults aged 65 years and older are roughly twice as likely to experience adverse drug events and seven times more likely to be hospitalised for them than younger adults, placing a substantial and rising burden on health systems.

**Public health significance—Why is this work of significance to public health?**

- Reframing ADRs as dynamic trajectories rather than isolated events moves population-level prevention upstream, toward the modifiable conditions—polypharmacy accumulation, anticholinergic burden, prescribing cascades, and frailty—that drive escalation toward severe, costly outcomes.
- Up to two-thirds of ADR-related hospitalisations are potentially preventable through systematic medication review, deprescribing, and frailty-informed prescribing, representing a large and avoidable share of drug-related harm and health-system expenditure.

**Public health implications—What are the key implications or messages for practitioners, policy makers and/or researchers in public health?**

- For practitioners: embed comprehensive geriatric assessment, STOPP/START version 3 and Beers-guided review, and structured deprescribing into the routine care of older adults, intervening at the amplifier stage before sentinel events occur.
- For researchers: develop and validate trajectory-sensitive outcome measures and AI-enabled frailty and ADR-risk prediction tools, and design longer trials that capture functional and patient-reported endpoints meaningful to older populations.

**Abstract:**

Background: Adverse drug reactions (ADRs) represent a major and often underestimated source of morbidity, hospitalization, and functional decline in older adults. The convergence of age-related pharmacokinetic and pharmacodynamic changes, multimorbidity, polypharmacy, and frailty creates a clinical environment in which ADR risk is not static but evolves along progressive trajectories—from mild, early manifestations toward severe, potentially irreversible outcomes. Understanding these trajectories is essential for rational geriatric prescribing. Methods: This narrative review synthesizes evidence from epidemiological studies, systematic reviews, Cochrane analyses, and clinical trials published between 2000 and 2025, focusing on adults aged 65 years and older with two or more chronic conditions. Sources were identified through a structured, non-systematic literature search of PubMed, EMBASE, Cochrane Library, Web of Science, and Scopus using the terms ‘adverse drug reactions’, ‘polypharmacy’, ‘multimorbidity’, ‘frailty’, ‘deprescribing’, and ‘pharmacokinetics’ in older adults, alone and in combination. Evidence quality was assessed narratively, distinguishing trial evidence from observational and expert consensus data. Results: ADRs in older adults are best classified using complementary frameworks—the augmented Type A to withdrawal Type E and failure-of-therapy Type F taxonomy (Types A–F), the Dose-Time-Susceptibility (DoTS) classification, and the EIDOS mechanistic scheme—which together capture the heterogeneity of drug-related harm in this population. Age-related pharmacokinetic changes (altered absorption, increased volume of distribution of lipophilic drugs, reduced hepatic and renal clearance) and pharmacodynamic shifts (heightened receptor sensitivity, baroreflex impairment, increased blood–brain barrier permeability) interact with polypharmacy and frailty to amplify ADR trajectories from mild to severe. Anticholinergic burden, prescribing cascades, and inappropriate polypharmacy function as structural accelerators of these trajectories. Medication review and deprescribing improve prescribing quality but evidence for hard outcome benefits remains of low to very low certainty. Emerging AI-enabled digital tools show promising accuracy for identifying frailty and pharmacological vulnerability, but this performance relates to frailty classification and has not yet been shown to prevent ADR trajectories; they require validation for routine clinical use. Conclusions: Recognizing ADRs in older adults as dynamic trajectories rather than isolated events repositions prescribing review and deprescribing from optional to essential clinical acts. An integrated approach combining pharmacological vigilance, comprehensive geriatric assessment, structured deprescribing, and emerging digital decision-support tools offers the most realistic pathway to reduce the trajectory-related burden of drug-related harm in complex older patients.

## 1. Introduction

The global health landscape has undergone a fundamental transformation over recent decades. Seven of the ten leading causes of mortality worldwide are now chronic non-communicable diseases, and population ageing has compounded this shift by dramatically increasing the prevalence of multimorbidity—defined as the coexistence of two or more chronic conditions—in later life. Approximately 46–48% of older adults globally live with multimorbidity, a proportion rising to 83% among those aged 85 and older [1,2].

Within this context, pharmacotherapy plays a dual and paradoxical role. Therapeutic drugs can improve healthy ageing by preventing and managing diseases, slowing disease progression, and reducing the burden of disability. Yet they can simultaneously impair physical and cognitive function, disrupt social engagement, and—particularly in the presence of polypharmacy, multimorbidity, and frailty—become major sources of iatrogenic harm. This complex, multidirectional relationship between drugs and healthy ageing has been described by Hilmer, Ferrucci, and Cherubini as a foundational challenge of geriatric medicine: evidence for drug efficacy and safety in older adults is mostly derived from trials that exclude or under-represent this population, and that do not include functional or well-being outcomes [3]. In keeping with this view, in the older patient it is frailty status rather than chronological age that should anchor pharmacological risk stratification and prescribing decisions [3].

Adverse drug reactions (ADRs)—defined as any noxious, undesired, or unintended response to a therapeutic agent at dosages used for prophylaxis, diagnosis, or therapy—are a health priority in older adults. Approximately 5% of all hospital admissions are attributable to ADRs, and in the United States more than 700,000 emergency department visits annually involve older patients experiencing adverse drug events. Patients aged 65 and older are estimated to be twice as likely to experience adverse drug events and seven times more likely to require hospitalization compared to younger adults [4,5].

However, a static, event-centred view of ADRs is insufficient for older adults. In this population, ADR risk does not remain constant: it evolves over time, driven by the progressive accumulation of pharmacokinetic vulnerabilities, the expansion of polypharmacy, the deepening of frailty, and the compounding effect of prescribing cascades. ADRs in older adults are better understood as trajectories—temporal progressions from mild initial manifestations (dry mouth, mild sedation, early orthostasis) toward severe downstream outcomes (falls, delirium, acute kidney injury, aspiration pneumonia, arrhythmia)—that are shaped by the interaction between ageing biology, multimorbidity, and therapeutic complexity [3,6].

This narrative review proposes a trajectory-based framework for understanding ADRs in older adults, integrating pharmacological classification, biological mechanisms, clinical determinants, and evidence-based strategies for safer prescribing. It is addressed to geriatricians, internists, pharmacists, and all clinicians involved in the complex pharmacological management of older patients with multimorbidity and frailty.

## 2. Classification of Adverse Drug Reactions in Older Adults

The conceptual precision with which ADRs are classified has direct implications for clinical practice. A useful classification helps clinicians predict, detect, and prevent drug-related harm. Three complementary frameworks are most relevant for geriatric pharmacology [4].

The most widely used taxonomy was originally proposed by Rawlins and Thompson in 1977, initially distinguishing only two reaction types—Type A (augmented) and Type B (bizarre) [7]—and was subsequently expanded by later authors to the six-category scheme (Types A–F) now in common use [8]. Type A reactions are augmented, dose-related, predictable, and common—examples include orthostatic hypotension with antihypertensives, bleeding with anticoagulants, and anticholinergic effects with tricyclic antidepressants. In older adults, Type A reactions predominate and are especially dangerous because they arise from otherwise expected pharmacological effects amplified by age-related vulnerability. Type B reactions are bizarre, non-dose-related, unpredictable, and potentially fatal—such as anaphylaxis to penicillin or malignant hyperthermia. Type C reactions are cumulative and chronic—such as hypothalamic– pituitary–adrenal suppression with corticosteroids. Type D reactions are delayed, such as tardive dyskinesia. Type E reactions are end-of-treatment effects, such as withdrawal syndromes from benzodiazepines or beta-blockers. Type F reactions represent unexpected therapeutic failures, often related to drug interactions or non-adherence [8].

The Dose-Time-Susceptibility (DoTS) classification adds a clinically important dimension by incorporating the temporal course of the reaction and individual susceptibility factors—including age, frailty, comorbidity, and genetic polymorphisms—alongside dose-relatedness. This framework is particularly valuable in geriatrics because it makes susceptibility an explicit analytical category, rather than an implicit background variable [8].

The EIDOS classification analyses the biochemical mechanisms of ADRs by distinguishing: the Extrinsic chemical species initiating the effect, the Intrinsic species involved, their Distribution in the body, the Outcome at the site of action, and the final Sequela that constitutes the adverse reaction. Used alongside DoTS, EIDOS allows clinicians to reason mechanistically about why a given patient at a given moment is particularly vulnerable to a specific drug effect—a reasoning strategy that is essential when managing older adults on complex polypharmacy regimens [4].

In clinical practice, these classifications are complementary: DoTS identifies the pharmacological and temporal structure of the risk; EIDOS explains the underlying mechanism; and the Type A–F taxonomy guides management—from dose reduction and monitoring for Type A reactions, to mandatory withdrawal for Type B, to planned tapering for Type E withdrawal syndromes. In older adults, the majority of preventable ADRs belong to the Type A category and are therefore theoretically predictable and avoidable with careful clinical reasoning [4].

## 3. Pharmacokinetic and Pharmacodynamic Vulnerabilities in Ageing

Ageing is accompanied by a series of physiological changes that profoundly alter how drugs are absorbed, distributed, metabolized, and eliminated, as well as how the body responds to them at the receptor level. These changes create the biological substrate upon which ADR trajectories are amplified [6,9].

### 3.1. Pharmacokinetic Changes

Absorption of drugs may be altered by reduced gastric acidity and slower intestinal motility, leading to unpredictable plasma concentrations for drugs with pH-dependent absorption such as certain antibiotics and oral antifungals. Distribution is markedly affected by the characteristic body composition changes of ageing: total body fat mass increases (reaching approximately 25% in men and 40% in women over age 70, compared to 15–20% in young adults), while total body water decreases progressively. This shift increases the volume of distribution of lipophilic drugs such as benzodiazepines, extending their half-life and increasing the risk of accumulation, sedation, and confusion. Conversely, hydrophilic drugs have a reduced volume of distribution and may reach higher peak plasma concentrations than expected [6,9].

Hepatic metabolism typically decreases with age due to reduced liver mass (by 20–40%), reduced hepatic blood flow (by 40–60%), and a decline in cytochrome P450 oxidase activity of up to 30% in people over 70 [9]. Drugs that are extensively metabolized by the cytochrome P450 system—including statins, direct oral anticoagulants, and many psychotropic agents—may accumulate, increasing the risk of toxicity. Plasma protein binding is further modified by the 19% lower albumin levels typically observed in elderly populations [9], raising the free fraction and potential toxicity of highly protein-bound drugs even when total drug concentrations appear within therapeutic range. Renal excretion declines progressively with age due to reduced glomerular filtration and tubular secretion, extending the half-life of renally cleared drugs—including metformin, digoxin, and many antibiotics—and substantially raising the risk of dose-dependent toxicity [6,9].

### 3.2. Pharmacodynamic Changes

Pharmacodynamic changes in older adults are equally consequential. Receptor-level alterations can amplify or distort drug effects independently of plasma concentration. Of particular clinical importance is the heightened sensitivity of the central nervous system to sedatives, antipsychotics, opioids, and anticholinergic agents—a consequence of increased blood–brain barrier permeability, reduced central cholinergic reserve, and altered neurotransmitter dynamics. This manifests clinically as an increased propensity for delirium, cognitive impairment, and falls even at doses that would be considered modest in younger patients. Cardiovascular compensatory capacity is reduced, making older adults more vulnerable to drug-induced orthostatic hypotension—particularly with vasodilators, alpha-blockers, neuroleptics, and diuretics. Receptor alterations in the dopaminergic, serotonergic, and beta-adrenergic systems further modify the therapeutic and adverse effect profiles of many commonly used medications [6,9].

Frailty adds an additional layer of pharmacological vulnerability to these physiological changes. The accumulation of biological deficits that characterises frailty impairs organ reserve, reduces homeostatic capacity, and substantially increases the risk of drug-induced harm. Patients with a frailty index of 0.16 or greater have been shown to be twice as likely to develop at least one ADR during hospitalisation, and to experience potentially inappropriate prescribing as classified by STOPP criteria, compared to non-frail patients. Frailty also produces ‘atypical’ presentations of drug toxicity—falls, delirium, and functional decline—that may not be immediately recognized as medication-related, perpetuating delayed identification and further harm [3,4].

These pharmacokinetic and pharmacodynamic changes, when combined with the occurrence of new comorbidities and the progressive expansion of polypharmacy, produce what may be conceptualized as the first stage of an ADR trajectory: an environment of latent vulnerability in which even modest pharmacological exposures can trigger clinically significant harm.

## 4. Polypharmacy as a Structural Amplifier of ADR Trajectories

Polypharmacy—conventionally defined as the concurrent use of five or more medications—is intrinsic to the management of multimorbidity and should not be equated with inappropriate prescribing. When appropriate, the benefits of multiple medications clearly exceed their risks and are aligned with patient goals and preferences. In England, more than half of people aged 85 and over are prescribed five or more medications, and a quarter receive eight or more; multimorbidity affects approximately 85% of this age group. The convergence of these two phenomena—polypharmacy and multimorbidity—creates a clinical environment of extreme pharmacological complexity [10].

What matters clinically is not the pill count per se, but whether the combination of medications an individual patient receives is appropriate or inappropriate for their specific circumstances. Inappropriate polypharmacy occurs when prescribing is not evidence-based, when the likelihood of harm exceeds the likelihood of benefit, when hazardous drug combinations or interactions are present, when the patient experiences unacceptable therapeutic burden, when adherence is reduced, or when prescribing cascades are in operation [10].

The prescribing cascade is among the most clinically dangerous manifestations of inappropriate polypharmacy. It occurs when an ADR is misinterpreted as a new medical condition, leading to the prescription of an additional drug to treat the apparent symptom, which in turn may cause further adverse effects. Classic examples include calcium channel blockers causing peripheral oedema misdiagnosed as heart failure and treated with a loop diuretic; cholinesterase inhibitors precipitating urinary incontinence misdiagnosed as overactive bladder and treated with a bladder anticholinergic; or antipsychotic-induced extrapyramidal symptoms treated with anti-parkinsonian drugs. In each case, the cascade compounds the initial drug burden, increases the total ADR risk, and generates a self-reinforcing cycle of increasing pharmacological complexity with diminishing clinical benefit [4,10,11].

Modern medicine is structured in a way that promotes polypharmacy accumulation but lacks equivalent mechanisms for its reduction. Clinical guidelines are largely organized around single-disease management and invariably promote prescribing; few include explicit deprescribing criteria. Barriers to deprescribing include the fear of negative consequences of stopping a perceived ‘life-saving’ medication, uncertainty about which drugs are causally beneficial, inadequate knowledge of evidence-based medicine, patient expectations of continued therapy, time constraints in clinical encounters, and suboptimal communication between multiple prescribers. Recognizing that polypharmacy is often necessary but that the rate of prescribing frequently outpaces the rate of deprescribing—creating a progressive, unreviewed accumulation of pharmacological burden—is a fundamental step toward trajectory-aware prescribing in older adults [10].

## 5. Anticholinergic Burden and Clinical Complications

Anticholinergic drugs—including tricyclic antidepressants, first-generation antihistamines, urinary antispasmodics, and many commonly used medications with incidental anticholinergic properties—represent one of the most clinically significant contributors to ADR trajectories in older adults. Their cumulative pharmacological effect, referred to as anticholinergic burden, disrupts cholinergic transmission in brain regions controlling alertness, attention, and memory, increasing the risk of delirium and accelerating cognitive decline. Systemically, anticholinergic medications reduce secretions and slow intestinal transit, contributing to dry mucous membranes, dysphagia, constipation, and urinary retention. In frail or cognitively impaired older adults, these effects may contribute to aspiration pneumonia, falls, and progressive functional decline [4,12].

Delirium is a paradigmatic sentinel outcome of the anticholinergic trajectory, and it is frequently missed when it is superimposed on pre-existing dementia. Because delirium is generally amenable to treatment once recognised, its detection imposes a clinical duty to investigate and address reversible precipitants, including drug-related ones. Validated bedside instruments now allow rapid identification of delirium superimposed on dementia—for example, a brief combined arousal-and-attention testing procedure [13] and short caregiver-administered triage questionnaires for outpatients with cognitive impairment [14]—and their routine use is consistent with a trajectory-aware approach to anticholinergic prescribing.

Observational evidence consistently associates higher anticholinergic burden with the development of cognitive decline or dementia in cognitively healthy older adults, and with more rapid deterioration in those with pre-existing cognitive problems. However, the interventional evidence for anticholinergic deprescribing as a strategy to improve cognitive outcomes is of very low certainty. A Cochrane systematic review by Taylor-Rowan and colleagues, examining three randomised controlled trials with a total of only 299 participants and follow-up periods of one to three months, found inconsistent results on cognitive outcomes and no between-group differences for any other clinical outcome, including falls, quality of life, functional impairment, and mortality. The review concluded that there is currently insufficient evidence from RCTs to support or refute the hypothesis that actively reducing anticholinergic burden improves cognitive outcomes in older adults, and that larger trials with longer follow-up and outcomes stratified by baseline cognitive status are urgently needed [12].

A further complexity in clinical practice is the substantial heterogeneity among the tools used to measure anticholinergic burden. The Anticholinergic Cognitive Burden Scale (ACB), the Anticholinergic Drug Scale (ADS), the Drug Burden Index (DBI), and other instruments include different medications, assign different scores, and are derived from different methodological approaches. Consequently, they may yield substantially different burden estimates for the same patient and different predictions of clinical risk. The DBI, which quantifies the total functional burden of medications with anticholinergic and sedative effects, has been associated with a wide range of adverse geriatric outcomes in international observational studies [12,15].

Despite this evidential uncertainty, clinical reasoning supports a cautious approach to anticholinergic prescribing in older adults. The plausibility of harm is strong, the observational evidence base is consistent, and many anticholinergic indications have effective alternatives with lower central nervous system burden. Minimising anticholinergic exposure—while acknowledging that deprescribing in practice may not always be feasible or immediately effective—remains a rational component of trajectory-aware pharmacological management.

## 6. Disease-Specific ADR Profiles in Older Adults

The interaction between guideline-recommended pharmacotherapy and the physiological vulnerabilities of older adults produces disease-specific ADR profiles that are qualitatively distinct from those observed in younger, non-frail patients. Table 1 summarises these profiles across ten major chronic diseases, distinguishing generic ADRs from those amplified or modified by ageing, frailty, and multimorbidity.

## 7. Frailty and Disability: Critical Distinctions for Pharmacological Risk Stratification

Frailty and disability are conceptually distinct but clinically overlapping states that modify pharmacological risk in different and important ways. Frailty—broadly understood as a state of increased vulnerability to stressors due to the accumulation of biological deficits and the depletion of physiological reserve—is not equivalent to disability, although the two conditions frequently coexist and may transition into one another [3,10].

Frailty operates as a pharmacokinetic and pharmacodynamic amplifier: it increases drug half-lives through impaired renal and hepatic clearance, reduces homeostatic capacity to buffer adverse effects, increases blood–brain barrier permeability to centrally acting drugs, and predisposes patients to atypical presentations of drug toxicity—falls, delirium, and functional decline—that may not be immediately recognised as medication-related. Disability, by contrast, reflects functional limitation that may arise from frailty but may also derive from fixed organ damage, structural neurological disease, or musculoskeletal conditions. Disability does not in itself necessarily increase pharmacological vulnerability in the same mechanistic sense that frailty does [3,10].

This distinction matters for prescribing decisions. A patient with severe physical disability due to established stroke who is otherwise robustly physiological may tolerate therapeutic doses of anticoagulants, antihypertensives, and antidepressants without significant amplification of ADR risk. The same level of apparent functional limitation in a frail, sarcopenic older adult with borderline renal function, low albumin, and polypharmacy represents a fundamentally different pharmacological risk profile, where the trajectory from mild to severe ADR may be compressed and accelerated [3].

Several established tools can help quantify frailty for pharmacological risk stratification, including the Clinical Frailty Scale, the Fried frailty phenotype, the frailty index derived from Comprehensive Geriatric Assessment, and STOPP/START version 3 criteria. The STOPP/START version 3 criteria represent the most current validated tool for identifying potentially inappropriate prescribing and prescribing omissions in older adults, and should be applied consistently as part of medication review in frail patients [26].

Practitioners should also consider that frailty is not a static state: it may improve with appropriate nutritional, rehabilitation, and pharmacological management, and this improvement may in turn alter the risk–benefit balance of specific medications. Trajectory-aware pharmacological management therefore requires repeated frailty assessment over time, not a single snapshot at the point of diagnosis or hospital admission.

## 8. Strategies for Safer Prescribing and Deprescribing

### 8.1. Medication Review and Medicines Optimisation

Medicines optimisation—the systematic process of improving prescribing quality for individual patients—is the cornerstone of ADR trajectory reduction in older adults. It encompasses more than a simple medication review or an audit of potentially inappropriate prescriptions. It is a patient-centred, iterative process that requires three stages: first, gathering background information (accurate medication list, indications and duration of therapy, frailty status, disease trajectory, and life expectancy); second, a structured discussion with the patient exploring their goals, therapeutic burden, experience of adverse effects, and medication adherence; and third, implementing agreed changes, communicating them to all prescribers involved, and arranging close follow-up [10].

The evidence base for medication review and pharmaceutical care interventions in older adults with polypharmacy has been extensively evaluated in a Cochrane systematic review by Cole and colleagues, which identified 38 randomised and cluster-randomised trials encompassing 24 study designs and a variety of settings. The overall conclusion was that pharmaceutical care may slightly reduce potentially inappropriate medications and prescribing omissions, but the certainty of evidence for all primary outcomes ranged from low to very low, reflecting high risk of bias and substantial heterogeneity across studies. No consistent evidence was found that these interventions improve major patient outcomes such as falls, hospitalisations, or mortality [27].

The effectiveness of medication review also depends critically on who performs it. Evidence from the UK HOMER randomised controlled trial of home-based medication review indicates that managing complex, frail patients requires advanced clinical training and experience, and that such interventions do not perform well when delivered by entry-level staff [28]; this argues for embedding medicines optimisation within specialist geriatric expertise rather than treating it as a generic, protocol-driven task.

These results should not be interpreted as evidence of futility, but rather as a reflection of the methodological challenges inherent in studying complex, multifaceted interventions in heterogeneous older populations. The appropriate response is not to abandon medication review, but to conduct it more systematically, to pair it with non-pharmacological alternatives for conditions often managed pharmacologically, and to measure outcomes that are meaningful to patients—including quality of life, therapeutic burden, and symptom control—rather than relying exclusively on administrative or hospitalisation endpoints [10,27].

### 8.2. Deprescribing

Deprescribing—the planned, supervised process of reducing or stopping medications for which the balance of harm and benefit has shifted unfavourably—is a core therapeutic act in geriatric medicine, not a peripheral consideration. The clinical rationale is compelling: older adults with multimorbidity accumulate medications over decades, clinical trial evidence supporting many of these prescriptions was generated in younger, healthier populations, and the life expectancy context may render preventive medications less meaningful relative to their burden [10].

Medications that most frequently warrant deprescribing consideration in older adults include: medications whose duration of benefit has been exceeded (bisphosphonates, proton pump inhibitors, antidepressants beyond the recommended period); drugs for which the evidence base has changed (beta-blockers following myocardial infarction with preserved ejection fraction); prescribing cascade-driven additions (loop diuretics added for calcium channel blocker-induced oedema; bladder anticholinergics added for cholinesterase inhibitor-induced urgency); and high-risk medications such as anticholinergics, sedatives, antipsychotics, and NSAIDs, for which the risk of harm in older adults is well established [10].

In practice, however, the gap between deprescribing recommendations and their implementation is substantial. Clinicians frequently encounter strong prescriber inertia—captured by the common objection that a patient “has taken it for twenty years without apparent harm”—and a justified fear of the “domino effect,” whereby withdrawing one medication in a frail patient destabilises an apparently balanced regimen. These concerns often lead to deprescribing decisions being deferred or referred back to community settings that lack the time and resources to manage them safely. Acknowledging these real-world barriers is essential: a trajectory-aware framework is only useful if it is paired with realistic strategies and adequately resourced clinical settings for acting on it.

Effective deprescribing requires a shared decision-making approach: creating awareness that options exist (prescribing, deprescribing, non-drug alternatives, or doing nothing); discussing the potential benefits and risks of each option; exploring patient preferences; and making the decision together. A validated five-step deprescribing protocol—encompassing systematic medicine revision, overall risk–benefit assessment for each drug, identification of drugs to discontinue, planned cessation with monitoring for withdrawal effects, and close follow-up—provides a practical operational structure for this process. Labelling the process as a ‘drug holiday’ or ‘trial without’ may improve patient understanding and willingness to participate. Some drugs can be stopped abruptly; others—notably long-term benzodiazepines, opioids, and antidepressants—require gradual dose reduction with careful monitoring for withdrawal effects or symptom recurrence [10,29].

### 8.3. Validated Prescribing Tools

Several validated tools support the deprescribing and medication optimisation process. The STOPP (Screening Tool of Older Persons’ Potentially Inappropriate Prescriptions) and START (Screening Tool to Alert doctors to the Right Treatment) criteria, now in their third version, provide a comprehensive, validated checklist of inappropriate prescriptions and important prescribing omissions in older adults, applicable as a clinical and research tool [26]. The Beers Criteria, published by the American Geriatrics Society and most recently updated in 2023, similarly list medications that are generally inappropriate for older adults due to high risk of adverse effects or better alternatives [19]. The FORTA (Fit fOR The Aged) list classifies drugs by their age-appropriateness across four categories from ‘absolutely indicated’ to ‘avoid’ [4].

No single tool comprehensively addresses all aspects of medicines optimisation—they differ in scope, methodology, and the degree to which they promote individualised clinical judgment. Their greatest value is as screening instruments to identify older patients at high risk of suboptimal pharmacotherapy and to prioritise areas for review, rather than as definitive quality measures. Their effectiveness is maximised when embedded within a broader, patient-centred clinical encounter rather than applied as a checklist in isolation [4,27].

### 8.4. Comprehensive Geriatric Assessment

The most evidence-supported context for ADR reduction in older adults is the Comprehensive Geriatric Assessment (CGA)—a multidisciplinary, multidimensional evaluation of medical, functional, cognitive, psychological, social, and nutritional status. A large study of 834 frail older adults demonstrated that a CGA-based approach combined with systemic medication review produced a 35% reduction in serious ADRs and inappropriate drug use [30]. CGA enables the creation of multidimensional care plans for individual patients, avoids fragmented or poorly coordinated care, defines treatment priorities, and positions medication management within the context of the patient’s complete clinical and functional profile [4].

## 9. Economic Impact of ADRs in Older Adults

The economic consequences of ADRs in older adults extend well beyond the costs of immediate hospital management. Older illustrative estimates have placed the cost of medical errors and drug side effects in the elderly US population at over 200 billion dollars annually; this figure should be regarded as an indicative, non-contemporaneous estimate rather than a precise current value. In European countries, ADR-related hospitalisations represent a significant and growing financial burden on national healthcare systems, contributing to emergency department visits, prolonged inpatient stays, readmissions, and the purchase of additional pharmacological products to manage drug-induced complications [9].

Approximately 5% of all hospital admissions are attributable to ADRs, and 5% of hospitalised patients will experience a new ADR during their hospital stay. It has been estimated that up to two-thirds of ADR-related hospitalisations could be prevented with more careful prescribing, systematic medication review, and structured follow-up. In two out of three cases, rehospitalisation due to drug-related harm represents a preventable event [4,9].

Medication review and deprescribing carry significant potential for cost reduction, although direct evidence for cost-effectiveness from randomised trials remains limited. The most robust economic argument for trajectory-aware prescribing is indirect: preventing a single episode of ADR-related hospitalisation, delirium, hip fracture, or acute kidney injury avoids not only the direct cost of acute care but also the substantial downstream costs of rehabilitation, long-term care placement, and persistent functional decline. In this sense, the economic case for geriatric pharmacovigilance mirrors the clinical case: early identification of trajectory risk is far more cost-effective than management of its consequences.

## 10. Molecular Mechanisms Driving ADR Trajectories in Older Adults

A mechanistic understanding of how specific drug classes produce harm in ageing biology is essential for anticipating cross-drug interactions and for designing safer pharmacological regimens. Table 2 summarises the primary molecular targets, ageing and frailty amplifiers, sentinel adverse drug reactions, and high-risk pharmacological synergies for fourteen major drug classes commonly prescribed to older adults.

Several cross-cutting mechanistic themes emerge from this overview. First, centrally acting drugs—anticholinergics, benzodiazepines, opioids, and antipsychotics—converge on impaired CNS function through mechanisms that are individually harmful but whose combined effects in polypharmacy generate additive or synergistic harm that is disproportionate to any single agent’s contribution. Second, drugs that alter fluid and electrolyte balance—diuretics, SGLT2 inhibitors, ACE inhibitors, ARBs, and NSAIDs—interact to produce multi-organ vulnerability (particularly to acute kidney injury, orthostatic hypotension, and electrolyte-driven arrhythmias) that is substantially amplified in the dehydration-prone, low-albumin, renally vulnerable older patient. Third, drugs affecting cardiac repolarization—antipsychotics, macrolides, fluoroquinolones, and class III antiarrhythmics—share a common mechanism of hERG channel blockade and create additive QT prolongation risk when co-prescribed in the context of electrolyte derangements common in frail patients.

Understanding these mechanistic convergences allows clinicians to anticipate dangerous pharmacological combinations before harm occurs, rather than recognizing them retrospectively. This mechanistic vigilance is a core component of trajectory-aware prescribing.

## 11. Future Directions: Digital Tools and Artificial Intelligence

The integration of digital decision-support tools and artificial intelligence into geriatric pharmacological management represents a promising but still early frontier. Software systems such as the SENATOR (Software ENgine for the Assessment and Optimisation of drug and non-drug Therapy in Older peRsons) engine, which applies STOPP/START criteria algorithmically to optimise prescriptions and reduce ADR risk in hospitalised older adults with multimorbidity and polypharmacy, demonstrated feasibility in a multinational randomised trial but, importantly, did not achieve significant reductions in ADR incidence, partly due to limited adherence by medical staff to the generated recommendations [4].

Artificial intelligence approaches—particularly machine learning algorithms including random forest, support vector machines, and deep learning models—have shown meaningful accuracy for the identification and diagnosis of frailty syndrome in older adults, with several models achieving accuracy, sensitivity, specificity, or area under the curve values of 90% or greater. A scoping review by Velazquez-Diaz and colleagues identified 26 studies using AI for frailty identification, showing that the combined use of clinical data (electronic medical histories and validated questionnaires) with non-clinical kinematic data from inertial sensors monitoring activities of daily living provided the highest diagnostic performance. Machine learning was the most frequently used AI approach, employed in 18 of 26 studies [31].

It is essential to interpret these performance metrics precisely. The reported accuracy, sensitivity, specificity, and AUC values of 90% or greater pertain to the identification and classification of frailty, not to the prevention of ADRs or the modification of ADR trajectories. No randomised evidence currently demonstrates that AI-based frailty identification translates into reduced ADR incidence or improved patient outcomes. These tools should therefore be regarded as a plausible future application of AI to trajectory-aware prescribing—a hypothesis to be tested—rather than a validated ADR-prevention strategy.

The implications for ADR trajectory management are nonetheless significant. If AI tools can reliably identify frailty status and physiological vulnerability in real time—drawing on electronic health records, sensor-based activity monitoring, and multimodal patient data—they may, in principle, offer the potential to flag patients at high risk of ADR trajectory escalation before clinical harm occurs, and to support more timely and personalised medication review decisions. AI should be conceptualised as a complementary decision-support tool operating alongside clinical judgment, rather than as a replacement for it; the performance of AI algorithms must be regularly monitored after integration into clinical practice, and data quality and privacy must be rigorously addressed [31].

Future research priorities include the development and validation of AI models specifically designed to predict ADR trajectory risk in older adults with multimorbidity and polypharmacy; the integration of pharmacokinetic parameters (renal function, hepatic markers, albumin, anticholinergic burden scores) with functional and frailty measures in predictive models; and the conduct of clinical trials testing AI-enabled prescribing support against usual care, using outcomes that are meaningful to older patients—including quality of life, functional independence, and freedom from therapeutic burden. Novel gerotherapeutics that target the biology of ageing itself—including senolytics, senomorphics, and metformin repurposed for its AMPK-activating properties—may in the future modify the pharmacokinetic and pharmacodynamic landscape within which ADR trajectories develop, though clinical evidence for this remains preliminary [3].

## 12. Discussion

**Figure 1:**
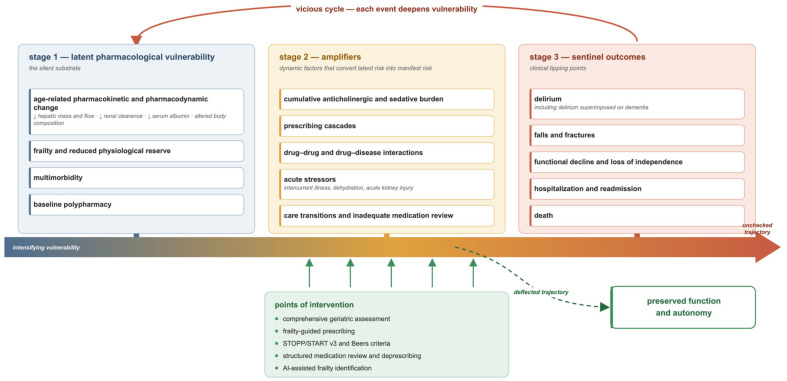
The adverse drug reaction trajectory in older adults. Schematic showing the three sequential phases—latent pharmacological vulnerability, dynamic amplifiers, and sentinel clinical outcomes—connected by a self-reinforcing feedback loop, together with the points of clinical intervention (comprehensive geriatric assessment, frailty-guided prescribing, STOPP/START version 3 and Beers criteria, structured medication review and deprescribing, and AI-assisted frailty identification) that can deflect the trajectory toward preserved function and autonomy.

We define an ADR trajectory as the time-dependent, directional progression of an individual older patient’s risk of drug-related harm, structured in three sequential phases—latent pharmacological vulnerability, dynamic amplifiers, and sentinel clinical outcomes—and characterised by a self-reinforcing feedback loop in which each sentinel event (a fall, an episode of delirium, an acute kidney injury) further deepens vulnerability and accelerates subsequent harm. So defined, the trajectory is distinct from several adjacent constructs with which it might be confused. It differs from a preventable ADR, which is a discrete event rather than a temporal progression; from a prescribing cascade, which is one amplifier mechanism operating within the trajectory rather than the trajectory itself; from cumulative drug burden (for example, anticholinergic burden indices), which is a static, cross-sectional measure whereas the trajectory is dynamic and directional; from frailty-related vulnerability, which constitutes the substrate of the trajectory rather than its full structure; and from longitudinal medication review, which is a recurring clinical intervention acting upon the trajectory rather than the phenomenon being acted upon. Figure 1 illustrates this trajectory and the points at which clinical intervention can deflect it.

The central proposition of this review is that ADRs in older adults are not isolated events but dynamic trajectories whose clinical significance is shaped by time, biology, and therapeutic complexity. This perspective has three practical implications that distinguish it from conventional ADR frameworks.

First, it repositions the point of clinical intervention. In a static, event-centred model, the clinician acts when an ADR has already occurred. In a trajectory-centred model, the clinician acts when the conditions for ADR escalation are being established—at the point of polypharmacy accumulation, frailty progression, or the first appearance of mild precursor symptoms (early orthostasis, subtle cognitive slowing, emerging constipation). This earlier intervention window is where pharmacological harm is most reversible and where the ratio of benefit to cost of intervention is most favourable [3,4].

Second, it reshapes how prescribing quality is evaluated. The standard metrics of inappropriate prescribing (Beers, STOPP) and prescribing omissions (START) describe a cross-sectional snapshot of the medication list at a given moment. A trajectory framework adds a longitudinal dimension: it asks not only whether a drug is inappropriate today, but whether the current pharmacological environment is creating conditions for harm escalation in the weeks or months ahead. This requires attention to trends—a rising creatinine, an increasing anticholinergic burden, a progressive decline in albumin, a new prescribing cascade—rather than just to the current clinical state [4,27].

Third, it changes the standard for evidence in geriatric pharmacology. The low certainty evidence from Cochrane reviews of medication review interventions and anticholinergic deprescribing does not reflect the ineffectiveness of these strategies; it reflects the inadequacy of current trial designs for capturing their benefits in complex older populations with heterogeneous multimorbidity, variable frailty, and outcome profiles that extend over years rather than months. Trajectory-sensitive trials—longer, broader in their outcome measurement, and inclusive of functional, patient-reported, and caregiver-centred endpoints—are needed to generate the evidence base that will meaningfully guide practice [12,27].

### Strengths and Limitations

This review offers a comprehensive synthesis across multiple dimensions of geriatric pharmacology—classification, biology, epidemiology, clinical profiles, and strategies—and is explicitly grounded in the most current and highest-quality available evidence, including recent Cochrane systematic reviews, validated prescribing tools at their latest iteration (STOPP/START version 3), and emerging evidence on digital decision support. It introduces a conceptual framework—ADR trajectories—that bridges mechanistic understanding and clinical practice in a way that is directly usable at the bedside.

Its primary limitation is the narrative, non-systematic format, which carries an inherent risk of selection bias in the choice and presentation of evidence; this review was conducted as a Perspective and does not apply the structured screening, formal inclusion/exclusion criteria, or risk-of-bias appraisal of a systematic review. The field of geriatric pharmacology is evolving rapidly and some specific recommendations may require updating as new trial evidence emerges. Most available evidence derives from high-income countries, limiting generalizability to settings with different healthcare structures, prescribing cultures, and patient demographics. The framework provides general principles and cannot substitute for individualized clinical judgment, which remains essential in the management of every complex older patient.

## 13. Conclusions

Adverse drug reactions in older adults should be understood as dynamic trajectories shaped by the interaction of ageing biology, multimorbidity, polypharmacy, and frailty—not as isolated events that occur unpredictably and without warning. The pharmacokinetic and pharmacodynamic vulnerabilities of ageing create a substrate of latent risk; polypharmacy, prescribing cascades, and anticholinergic burden function as amplifiers of that risk; and frailty compresses the clinical distance between mild precursor symptoms and severe outcomes.

Trajectory-aware geriatric pharmacology requires three parallel commitments. The first is mechanistic vigilance: understanding how specific drug classes interact with ageing biology and with each other to produce sentinel outcomes—delirium, falls, acute kidney injury, bleeding, cardiac arrhythmia, and aspiration pneumonia—that are predictable and largely preventable. The second is systematic prescribing review: embedding medicines optimisation, STOPP/START-guided assessment, and structured deprescribing into routine geriatric care, guided by patient goals, frailty status, life expectancy, and comprehensive multidisciplinary evaluation. The third is methodological humility: recognising that the evidence base for many pharmacological decisions in older adults is derived from trials that excluded this population, and that the certainty of evidence for many deprescribing and medication review interventions remains low—which calls for greater clinical conservatism, more rigorous and longitudinal trial designs, and the development of trajectory-sensitive outcome measures that capture what matters most to older patients.

Ultimately, the goal is not simply to count and reduce medications, but to align the pharmacological environment with the physiological reality of each older patient—reducing the burden of drug-related harm while preserving the therapeutic benefit that medications can and do provide for healthy ageing and functional independence.

## Figures and Tables

**Table 1:** Disease-specific ADR profiles: examples of commonly used, guideline-recommended therapies and their generic versus older-adult-specific side effects in major chronic diseases.

Chronic Disease	Guideline (Ref.)	First-Line Drugs (Examples)	Generic ADRs	Older-Adult–Specific ADRs
Heart Failure (HFrEF)	ESC 2023 [16]	ARNI (sacubitril/valsartan), beta-blockers (bisoprolol), MRA (spironolactone), SGLT2i (empagliflozin), loop diuretics	ARNI: hypotension, hyperkalaemia. Beta-blockers: bradycardia, fatigue. MRA: hyperkalaemia, gynaecomastia. SGLT2i: UTIs, dehydration. Diuretics: electrolyte imbalances	ARNI: heightened renal decline in frailty; severe hypotension. Beta-blockers: bradyarrhythmias, falls. MRA: severe hyperkalaemia in CKD. SGLT2i: orthostatic hypotension, euglycaemic ketoacidosis. Diuretics: dehydration, confusion/delirium
Stroke/Vascular Prevention	AHA/ASA 2021 [17]	Statins (rosuvastatin), antiplatelets (aspirin, clopidogrel), DOACs (apixaban)	Statins: myopathy, elevated LFTs. Antiplatelets: GI bleeding. DOACs: bleeding risk	Statins: myopathy risk increased; CYP interactions. Antiplatelets: amplified bleeding risk with falls. DOACs: accumulation in renal dysfunction
COPD	GOLD 2024 [18]	LAMA (tiotropium), LABA (salmeterol), ICS (eosinophilic phenotype)	LAMA: dry mouth, urinary retention. LABA: palpitations, tremor. ICS: oral candidiasis, hoarseness	LAMA: increased anticholinergic load; narrow-angle glaucoma. LABA: arrhythmias, tachyarrhythmias. ICS: heightened pneumonia risk, potential bone loss
Alzheimer’s Disease/BPSD	AGS Beers 2023 [19]	Acetylcholinesterase inhibitors (donepezil, rivastigmine), memantine, atypical antipsychotics (risperidone), antidepressants	Donepezil: nausea, bradycardia. Memantine: confusion, dizziness. Antipsychotics: EPS, sedation, QT prolongation. Antidepressants: sedation (TCAs)	Donepezil: syncope, conduction blocks. Memantine: delirium in frail patients. Antipsychotics: stroke risk, increased mortality in dementia. TCAs: anticholinergic effects (confusion, urinary retention)
Chronic Kidney Disease	KDIGO 2024 [20]	ACE inhibitors (ramipril), ARBs (losartan), SGLT2i (dapagliflozin), statins	ACE inhibitors: cough, hyperkalaemia. ARBs: hyperkalaemia, renal dysfunction. SGLT2i: UTIs, dehydration. Statins: myopathy	ACE inhibitors: AKI, orthostatic hypotension. ARBs: synergy with RAAS blockers → severe hyperkalaemia. SGLT2i: hypotension, falls. Statins: muscle weakness, polypharmacy interactions
Lower Respiratory Infections	ATS/IDSA 2019 [21]	Beta-lactams (amoxicillin ± clavulanate), macrolides (clarithromycin), fluoroquinolones (levofloxacin)	Beta-lactams: allergic reactions, diarrhoea. Macrolides: QT prolongation, GI upset. Fluoroquinolones: tendinopathy, neuropathy	Beta-lactams: confusion/delirium in frail older adults, renal dose adjustment required. Macrolides: arrhythmias, CYP3A4 interactions. Fluoroquinolones: severe delirium, tendon rupture, seizures in predisposed patients
Hypertension	ESC 2024 [22]	ACE inhibitors (lisinopril), ARBs (candesartan), CCB (amlodipine), thiazides (chlorthalidone), beta-blockers (bisoprolol)	ACE inhibitors: hypotension, cough. ARBs: dizziness, hyperkalaemia. CCB: peripheral oedema, constipation. Thiazides: hypokalaemia, dehydration. Beta-blockers: fatigue, bradycardia	ACE inhibitors: renal function decline in frail patients. ARBs: falls from dizziness. CCB: orthostatic hypotension, oedema worse in heart failure. Thiazides: hyponatraemia, fall risk. Beta-blockers: bradycardia, masking of hypoglycaemia
Lung Cancer	ESMO 2018 [23]	Platinum-based chemotherapy (cisplatin), EGFR inhibitors, immunotherapies (pembrolizumab), opioid analgesics	Cisplatin: nephrotoxicity, nausea. EGFR inhibitors: rash, diarrhoea. Immunotherapy: autoimmune reactions. Opioids: sedation, constipation, respiratory depression	Cisplatin: severe nephrotoxicity, electrolyte imbalances. EGFR inhibitors: severe diarrhoea, rash. Immunotherapy: atypical immune events in frail patients. Opioids: delirium, falls, synergy with anticholinergic burden
Type 2 Diabetes	ADA 2024 [24]	Metformin, SGLT2i (empagliflozin), GLP-1 RAs (liraglutide), insulin	Metformin: GI upset, lactic acidosis. SGLT2i: UTIs, dehydration. GLP-1 RAs: nausea, weight loss. Insulin: hypoglycaemia	Metformin: caution in renal impairment, lactic acidosis risk. SGLT2i: postural hypotension, euglycaemic ketoacidosis. GLP-1 RAs: exacerbated anorexia, malnutrition in frail patients. Insulin: severe hypoglycaemia in cognitively impaired or malnourished patients
Liver Cirrhosis	EASL 2018 [25]	Beta-blockers (propranolol), lactulose, rifaximin, diuretics (spironolactone), terlipressin	Beta-blockers: bradycardia, hypotension. Lactulose: diarrhoea, bloating. Rifaximin: GI discomfort, fatigue. Diuretics: electrolyte imbalances. Terlipressin: vasoconstriction, ischaemia	Beta-blockers: bradyarrhythmias with conduction disease. Lactulose: dehydration, confusion. Rifaximin: delirium in frail patients. Diuretics: AKI, hyperkalaemia, falls. Terlipressin: peripheral ischaemia, caution in cardiovascular disease

Note: The drugs listed are illustrative examples of commonly used, guideline-recommended therapies, not an exhaustive or definitive first-line list; the cited guideline for each condition is the most recent relevant guidance. Older-adult-specific side effects underscore how age-related physiological changes and polypharmacy can amplify or qualitatively modify typical adverse effects. Drug selection must be individualised based on frailty status, cognitive function, renal and hepatic reserve, comorbidity burden, and patient goals. Abbreviations: ADR: Adverse Drug Reaction; ARNI: Angiotensin Receptor-Neprilysin Inhibitors; MRA: Mineralocorticoid receptor antagonists; UTIs: Urinary tract infections; CKD: Chronic Kidney Disease; DOAC: Direct Oral Anticoagulants; LFTs: Liver Function Tests; CYP: Cytochrome P450; LAMA: Long-Acting Muscarinic Antagonist; LABA: Long-Acting Beta2-Agonist; ICS: Inhaled Corticosteroids; TCAs: Tricyclic antidepressants; ACE: Angiotensin-Converting Enzyme; RAAS: Inhibitors of the Renin-Angiotensin-Aldosterone System; CYP3A4: Cytochrome P-450 3A4; CCB: Calcium channel blockers; EGFR: Epidermal Growth Factor Receptor; GLP-1 RAs: GLP-1 receptor agonists.

**Table 2:** Molecular mechanisms of class-specific adverse drug reactions in older adults, with ageing/frailty amplifiers and high-risk pharmacological synergies. The mechanistic targets, amplifiers, and drug–drug interaction synergies summarised below are drawn from current geriatric-pharmacology and prescribing-safety sources, principally references [4,15,19,26].

Drug Class	Primary Target	Ageing/Frailty Amplifiers	Sentinel ADR(s)	High-Risk Synergies
Anticholinergics (TCAs, 1st-gen H1, bladder antispasmodics)	Muscarinic M1/M2/M3 antagonism	BBB leakiness; ↓ cortical cholinergic reserve; ↓ hepatic clearance; active metabolites (long t½)	Delirium, cognitive impairment, urinary retention, aspiration risk	Anticholinergic burden additive; opioids; benzodiazepines; sedating antihistamines
Benzodiazepines	GABAA positive allosteric modulation (α1/α5)	↑ Vd (lipophilicity); ↓ hepatic clearance; ↑ α5 sensitivity; P-gp downshift	Sedation, ataxia, falls, cognitive slowing, delirium	Opioids, Z-drugs, antipsychotics; antihypertensives/diuretics (orthostasis)
Antipsychotics	D2 antagonism ± H1/M1/α1	↓ Dopaminergic reserve; baroreflex impairment; QT substrate	Parkinsonism/EPS, orthostatic hypotension, QT prolongation, syncope	Macrolides/fluoroquinolones (QT); diuretics (hypoK/Mg); multiple QT drugs
SSRI/SNRI	SERT/NET inhibition; platelet SERT block	Frailty vasculopathy; polytherapy with antithrombotics	Bleeding (GI, mucosal), hyponatraemia, falls	ASA, P2Y12 inhibitors, DOACs/warfarin; CYP inhibitors (↑ exposure)
QT-prolonging agents (antipsychotics, macrolides, class III antiarrhythmics)	hERG/KCNH2 (IKr) blockade	HypoK/hypoMg (loop/thiazides), bradycardia, structural heart disease	Torsades de pointes, syncope	Diuretics (electrolyte loss); digoxin (arrhythmic substrate); multiple QT drugs
Beta-blockers	β1/β2 antagonism	↓ Sympathetic tone; baroreflex impairment; conduction disease	Bradycardia, AV block, bronchospasm, orthostatic symptoms	Non-DHP CCBs, digoxin (AV block); clonidine withdrawal
ACE inhibitors/ARBs	ACE inhibition → ↑ bradykinin/AT1 receptor antagonism	Subclinical hypovolaemia; RAAS hyporesponsiveness; CKD	Cough, angioedema, hypotension, AKI	MRA/ARB/trimethoprim (hyperK); NSAID (AKI ‘triple whammy’)
Loop diuretics	NKCC2 inhibition (TAL)	Frailty-related volume depletion; poor intake; CKD	Hypovolaemia, hypoK/Mg, AKI, ototoxicity	Digoxin (arrhythmias with hypoK); QT drugs; SGLT2i (volume)
SGLT2 inhibitors	SGLT2 block (proximal tubule)	Dehydration; low-carb intake; insulinopenia; CKD	Volume depletion, hypotension; euglycaemic ketoacidosis (rare)	Loop diuretics; ACE-i/ARB (AKI risk in hypovolaemia)
Statins	HMG-CoA reductase inhibition	Sarcopenia; SLCO1B1 variants; CYP3A4 interactions	Myalgia/myopathy, ↑ CK, rare rhabdomyolysis	Macrolides/azoles (CYP3A4); gemfibrozil; cyclosporine
Anticoagulants—DOACs	FXa/FIIa inhibition; P-gp/CYP3A4 substrate	CKD; P-gp/CYP3A4 inhibitors; low body weight	Major bleeding	Azoles/macrolides, amiodarone, verapamil/diltiazem (↑ levels)
NSAIDs	COX-1/COX-2 inhibition	CKD; RAAS blockers; dehydration	GI ulcer/bleed, AKI	‘Triple whammy’ (ACE-i/ARB + diuretic + NSAID); anticoagulants/antiplatelets
Opioids	μ/κ/δ opioid receptors	↓ Clearance in renal/hepatic dysfunction; constipation; opioid sensitivity ↑ with frailty	Sedation, respiratory depression, delirium, constipation, falls	Benzodiazepines (respiratory depression); anticholinergics (constipation, delirium)
Inhaled COPD agents (LAMA/LABA/ICS)	M3, β2, GR	Prostatic enlargement/glaucoma (LAMA); hypoK (LABA + diuretics); frailty (ICS pneumonia)	Urinary retention/glaucoma (LAMA); tachyarrhythmias (LABA); pneumonia (ICS)	Loop/thiazide diuretics (hypoK with LABA); chronic ICS + diabetes

Abbreviations: BBB, blood–brain barrier; P-gp, P-glycoprotein; RAAS, renin–angiotensin–aldosterone system; CKD, chronic kidney disease; EPS, extrapyramidal symptoms; QT, QT interval; TAL, thick ascending limb; Vd, volume of distribution, ↑ increased activity; ↓ reduced activity; → unchanged activity.

## Data Availability

No new data were created or analyzed in this study.
